# The influence of LncRNA H19 polymorphic variants on susceptibility to cancer: A systematic review and updated meta-analysis of 28 case-control studies

**DOI:** 10.1371/journal.pone.0254943

**Published:** 2021-07-26

**Authors:** Kunpeng Wang, Zheng Zhu, Yiqiu Wang, Dayuan Zong, Peng Xue, Jinbao Gu, Daoyuan Lu, Chuanquan Tu

**Affiliations:** 1 Department of Urology, The First People’s Hospital of Lianyungang, Lianyungang Clinical Medical College of Nanjing Medical University, Lianyungang, China; 2 First Clinical Medical College of Nanjing Medical University, Nanjing, China; 3 Department of Surgical Oncology, Xuzhou Central Hospital, Southeast University Cancer Institute, Xuzhou, China; Shanghai Jiao Tong University, CHINA

## Abstract

**Objective:**

Although myriad researches upon the associations between LncRNA H19 polymorphic variants (rs2839698 G>A, rs217727 G>A, rs2107425 C>T, rs2735971 A>G and rs3024270 C>G) and the susceptibility to cancer have been conducted, these results remained contradictory and perplexing. Basing on that, a systematic review and updated meta-analysis was performed to anticipate a fairly precise assessment about such associations.

**Methods:**

We retrieved the electronic databases EMBASE, PubMed and Web of Science for valuable academic studies before February 28, 2021. Ultimately, 28 of which were encompassed after screening in this meta-analysis, and the available data was extracted and integrated. The pooled odds ratios (ORs) with 95% confidence intervals (CIs) was used to evaluate such associations. For multi-level investigation, subgroup analysis derived from source of controls together with genotypic method was preformed.

**Results:**

Eventually, 28 articles altogether embodying 57 studies were included in this meta-analysis. The results illuminated that LncRNA H19 polymorphisms mentioned above were all irrelevant to cancer susceptibility. Nevertheless, crucial results were found concentrated in population-based control group when subgroup analysis by source of controls were performed in H19 mutation rs2839698 and rs2735971. Meanwhile, in the stratification analysis by genotypic method, apparent cancer risks were discovered by TaqMan method in H19 mutation rs2107425 and rs3024270. Then, trial sequential analysis demonstrated that the results about such associations were firm evidence of effect.

**Conclusion:**

Therefore, this meta-analysis indicated that LncRNA H19 polymorphisms were not associated with the susceptibility to human cancer. However, after the stratification analysis, inconsistent results still existed in different genotypic method and source of control. Thus, more high-quality studies on cancer patients of different factors were needed to confirm these findings.

## Introduction

As a major public health problem in the world, cancer is the second biggest cause of death in the developed countries. 1,762,450 new cancer cases and 606,880 cancer mortalities are predicted to occur in the United States in 2019 [1]. Nevertheless, the pathogeny of malignant tumor still remains vague. Consensus amongst worldwide researchers is that both the environmental and genetic abnormality contribute to the carcinogenesis [2]. The aberration of genetic expression increases the risk of the initiation and progression of cancer [3]. Accumulating studies have been focusing on the repercussions of long non-uncoding RNA (lncRNA) mutation which has a place in the genetic factors mentioned above [4–7].

LncRNA H19 is 2.3 kb in length, located on chromosome 11p15.5 and lacking the open reading frames [8]. The H19 gene, which is maternal imprinted and expressed, plays an irreplaceable role during embryonic phase and decreases in postpartum mature tissues [9]. As we know, LncRNA H19 is considered as a vital factor associated with, cancer susceptibility included, various biological process which impacts the invasion, metastasis, recurrence and poor prognosis of cancer [10]. It might extend the influence upon the development and progression of disease through the regulation of expression on and after transcription of the oncogene and antioncogene [11]. An increasing number of studies have revealed that H19 gene up-regulated in almost overall cancer, such as breast cancer, bladder cancer, colorectal cancer, gastric cancer, lung cancer, hepatocellular cnacer, ovarian cancer, pancreatic cancer and so on [4–7, 12].

Single nucleotide polymorphisms (SNPs), a type of genetic mutation, affect the gene expression and function, accordingly causing carcinogenesis [13]. Previous studies have indicated the associations between the risks of cancer and several SNPs (rs2839698 G>A, rs217727 G>A, rs2107425 C>T, rs2735971 A>G and rs3024270 C>G) [2, 14–18]. For instance, Wang et al. conducted a study and found that H19 polymorphism rs217727 might influence the susceptibility to non-small cell lung cancer (NSCLC) [19]. However, another study conducted by Lv et al. showed that H19 polymorphism rs217727 was not associated with overall cancer susceptibility [20]. In that case, though considerable researches have been performed, pooled results seem to be conflicting. Herein, this meta-analysis aimed at deriving a more accurate evaluation in all relevant published studies of the associations between the H19 SNPs and overall cancer susceptibility.

## Materials and methods

We conprehensively retrieved the electronic databases EMBASE, PubMed and Web of Science for all relevant articles published before February 28, 2021, utilizing terms including ‘H19 gene’, ‘polymorphisms’ or ‘genetic mutation’ with ‘LncRNA’ or ‘H19 SNPs’, and ‘cancer susceptibility’ or ‘tumor’. Potential eligible studies were collected and integrated by manual work. Additionally, we then removed the duplicate data from different articles.

Meanwhile, the remaining articles were screened by following criteria: (1) Independent case-control or cohort studies; (2) Possessing at least one of H19 polymorphisms (rs2839698 G>A, rs217727 G>A, rs2107425 C>T, rs2735971 A>G and rs3024270 C>G); (3) Availability of subgroup analysis statistical data of both cases and controls; (4) Enrolled patients with cancer diagnosed definitely by histopathological examination, and controls with no history of neoplasms. Correspondingly, the studies enrollment followed these exclusive criteria: (1) Without available data; (2) Without valuable results related to H19; (3) No case-control study.

### Data extraction

The available data from articles after screening were extracted and integrated respectively by two investigators (KP Wang and Z Zhu). Upon the appearance of divergence, a third investigator (YQ Wang) would take intervention and help make a better decision. All extracted data were integrated in an united form, especially with regard to the following information: First author’s name, Publication year, Ethnicity, Source of controls, Genotypic method, The number of cases and controls, The number of H19 polymorphisms carriers and non-carriers respectively as well as The results of the Hardy-Weinberg equilibrium (HWE) test.

### Statistical analysis

In the meta-analysis, the pooled odds ratios (ORs) with 95% confidence intervals (CIs) were used to estimate the strength of the associations between the H19 polymorphisms and cancer susceptibility, applying five main genetic comparison models: allele model, homozygous model, heterozygous model, dominant model and recessive model. According to Cochrane Q test and Higgins I [2] statistic, the fixed and random effect model were adopted. I2 < 50% suggested no obvious heterogeneity, in which case fixed effect model should be selected for calculation; only I2 ≧ 50% should the random effect model be selected. Generally, several factors, such as experimental scheme, sex, age, ethnicity, genotypic method and so on, could stimulate the heterogeneity. Therefore, subgroup analysis derived from source of controls and genotypic method was conducted, aiming at investigating the source of heterogeneity. The HWE test was adopted in the control groups to evaluate the gene and genotype frequency, and P value exceeded 0.05, guaranteeing a significant equilibrium. In addition, sensitive analysis was used to examine the stability and reliability of the results through recalculating the pooled ORs following the sequential exclusion of a single study at a time. Meanwhile, we conducted Begg’s funnel plots and Egger’s linear regression test in order to verify the publication bias among these studies Statistical data was analyzed through Stata statistical software (Version 12.0, Stata Corporation, College Station, TX, USA).

### Trial sequential analysis

The results of the meta-analysis should relate the total number of randomized participants accounting for statistical diversity to avoid type I errors. Thus, trial sequential analysis (TSA) was performed to estimate the required information size, which maintained a 95% confidence interval, a 20% relative risk reduction, an overall type I error of 5% and a statistical test power of 80%. TSA could confirm greater statistical data reliability through modifying the threshold with dispersive data for significance level. We then calculated the required information size and constructed the trial sequential monitoring boundaries. If the blue line (representing the cumulative Z-curve) cross the sloping red line (representing the trial sequential monitoring boundary) or exceed the vertical red line (representing the required information size), a significant result would be reached, and further studies will be unnecessary. On the contrary, either the information size required not being reached or the cumulative Z-curve not crossing the boundary reveals that additional clinical trials were necessary to reach the sufficient information size for further verification. The TSA software (TSA, version 0.9, 2011; Copenhagen Trial Unit, Copenhagen, Denmark) was adopted in this study.

## Results

### Studies characteristics

**Fig 1:**
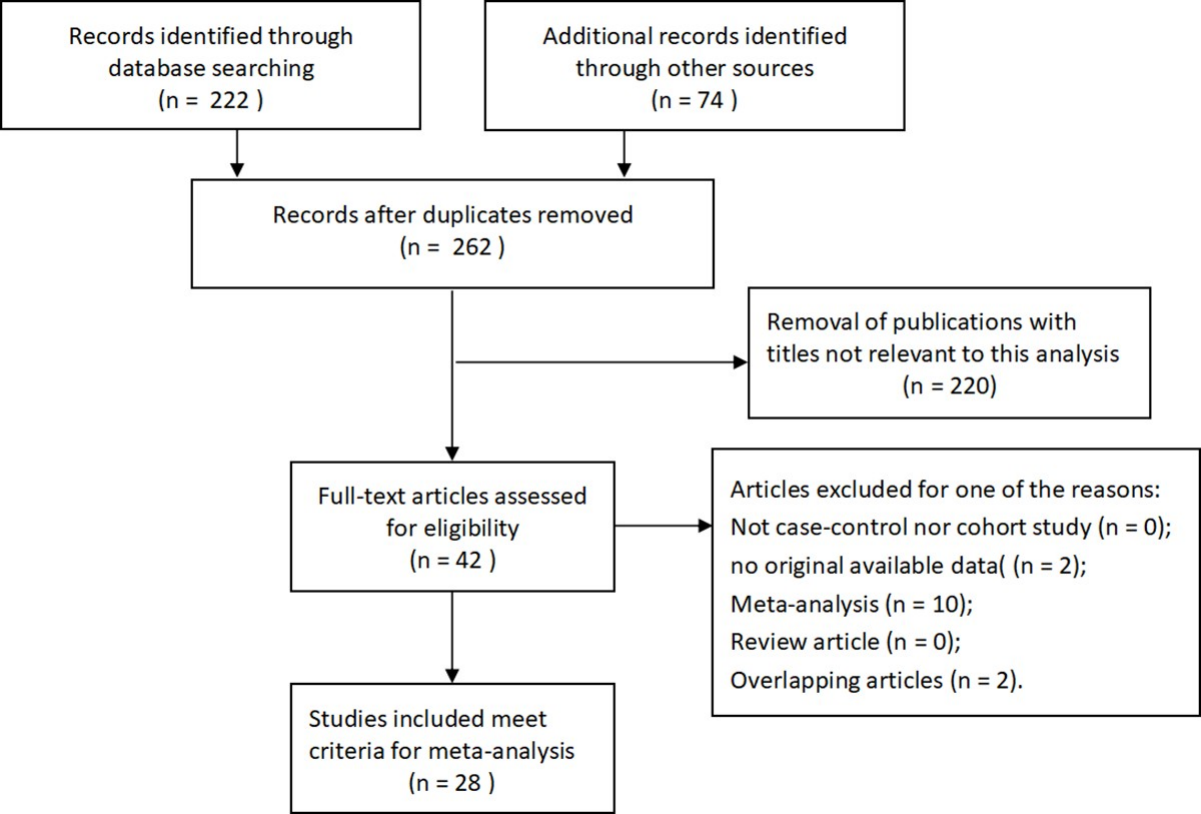
The flowchart of literature search and selection procedure.

Primitively, a total of 262 articles were collected under the guidance of the retrieve strategy above for further screening. Then, 28 articles containing 57 studies met the inclusive criteria, ranging from Feburary 2008 to February 2020 as for publish date [15–19, 21–33]. The flow pathway was shown in **Fig 1** [34–44]. Distribution of the genotypes in the controls was consistent in HWE. The baseline characteristics of all the studies in this meta-analysis were extracted and tabulated in **Table 1**. These studies involved Asians, Caucasians, Africans and Mixed. We separated these studies into two groups, including population-based group and hospital-based group, to help differentiate between various sources of control. Moreover, six genotypic methods altogether were performed in all these studies, such as Taqman, Illumina, PCR-RFLP, Sequenom and so on.

**Table 1:** Characteristics of individual studies included in the meta-analysis.

First author	Year	Country/Region	Racial	Source of controls	Case	Control	Genotype distribution						Genotyping methods	Test for HWE		Abbreviation
							case			control				χ^2^	*P*	
**16 Studies for *rs2839698* Poly morphism of the H19 Gene**																
							**GG**	**GA**	**AA**	**GG**	**GA**	**AA**				
Li	2016	China	Asian	PB	1147	1203	583	462	102	666	462	75	TaqMan	0.582	0.446	CRC
Hua	2016	China	Asian	HB	1049	1397	552	418	79	729	565	103	TaqMan	0.000	0.991	BC
Gong	2016	China	Asian	HB	496	206	237	220	39	99	80	27	TaqMan	1.515	0.218	LC
Yang	2015	China	Asian	HB	500	500	250	195	55	284	178	38	TaqMan	3.206	0.073	GC
Verhaegh	2008	Netherlands	Caucasian	PB	177	204	54	74	49	52	109	43	PCR-RFLP	4.713	0.030	BC
Yang	2018	China	Asian	HB	472	472	215	211	40	245	185	32	KASP	1.372	0.241	HCC
Guo	2017	China	Asian	HB	362	741	133	171	58	244	377	120	Illumina	0.060	0.806	OSCC
He	2017	China	Asian	HB	193	383	83	98	12	178	175	30	TaqMan	5.876	0.015	Osteosarcoma
Cui	2018	China	Asian	HB	1491	1677	801	568	122	875	673	129	TaqMan	2.238	0.135	BCa
Mohammad	2019	Iran	Asian	HB	111	130	15	57	39	53	55	22	4P-ARMSPCR	0.665	0.415	BCa
Wu	2019	China	Asian	HB	359	1190	140	178	41	532	524	134	TaqMan	1.927	0.165	HCC
Yang	2019	China	Asian	HB	431	431	206	170	55	192	184	55	PCR	4.376	0.036	BC
Wang	2019	China	Asian	HB	563	1532	277	225	61	712	645	175	TaqMan	2.217	0.136	LC
Lin	2017	China	Asian	HB	1005	1020	452	440	113	484	432	104	PCR	0.144	0.705	BCa
Yu	2020	China	Asian	HB	315	441	134	140	40	154	211	74	PCR	0.132	0.716	CRC
Zhang	2020	China	Asian	HB	201	196	70	93	38	92	88	16	Sequenom MassARRAY	0.514	0.473	OC
**17 Studies for *rs217727 G* Poly morphism of the H19 Gene**																
							**GG**	**GA**	**AA**	**GG**	**GA**	**AA**				
Hua	2016	China	Asian	HB	1046	1394	431	467	148	573	665	156	TaqMan	1.397	0.237	BC
Li	2016	China	Asian	PB	1147	1203	480	514	153	456	570	177	TaqMan	0.686	0.407	CRC
Xia	2016	China	Asian	PB	464	467	160	156	148	139	212	116	CRS-RFLP	0.490	0.000	BCa
Yang	2015	China	Asian	HB	500	500	160	252	88	193	244	63	TaqMan	0.431	0.512	GC
Verhaegh	2008	Netherlands	Caucasian	PB	177	204	114	59	4	115	80	9	PCR-RFLP	1.314	0.252	BC
Yin	2018	China	Asian	PB	556	395	204	264	88	165	172	58	Illumina	0.028	0.866	LC
Guo	2017	China	Asian	HB	362	740	101	181	80	255	348	137	Illumina	0.004	0.949	OSCC
He	2017	China	Asian	HB	193	383	79	102	12	195	165	23	TaqMan	7.862	0.005	Osteosarcoma
Abdollahzadeh	2018	Iran	Asian	HB	150	100	116	29	5	86	14	0	PCR-RFLP	3.167	0.075	BCa
Cui	2018	China	Asian	HB	1488	1675	611	692	185	685	773	217	TaqMan	0.257	0.612	BCa
Hu	2017	China	Asian	HB	416	416	133	200	83	128	196	92	TaqMan	0.247	0.619	PC
Mohammad	2019	Iran	Asian	HB	111	130	79	30	2	64	54	12	4P-ARMSPCR	0.195	0.659	BCa
Wu	2019	China	Asian	HB	359	1190	154	170	35	495	539	156	TaqMan	1.470	0.225	HCC
Yang	2019	China	Asian	HB	431	431	185	202	44	191	188	52	PCR	1.065	0.302	BC
Wang	2019	China	Asian	HB	564	1535	162	277	125	493	751	291	TaqMan	0.103	0.749	LC
Li	2019	China	Asian	HB	200	200	51	140	9	84	90	26	TaqMan	43.168	0.000	BC
Lin	2017	China	Asian	HB	1005	1020	403	471	131	465	450	105	PCR	0.131	0.718	BCa
Li	2018	China	Asian	HB	555	618	210	250	95	246	305	97	TaqMan	1.911	0.167	LC
**10 Studies for *rs2107425* Poly morphism of the H19 Gene**																
							**CC**	**CT**	**TT**	**CC**	**CT**	**TT**				
Verhaegh	2008	Netherlands	Caucasian	PB	177	204	92	65	20	89	96	19	PCR-RFLP	2.545	0.111	BC
Song	2009	Mixed	Caucasian	PB	5366	8538	2619	2192	555	4029	3667	842	TaqMan、Sequenom MassArray	9.082	0.003	OC
Quaye	2009	Mixed	Caucasian	PB	1460	2463	767	544	149	1118	1098	247	TaqMan	12.392	0.000	OC
Barnholtz-Sloan 11	2014	USA	Caucasian	PB	1225	1118	604	516	105	521	478	119	Illumina	0.124	0.725	BCa
Barnholtz Sloan 12	2014	USA	Africa	PB	737	658	161	390	186	170	339	149	Illumina	2.615	0.106	BCa
Butt	2012	Sweden	Caucasian	PB	678	1355	360	250	68	637	573	145	Sequenom	6.067	0.014	BCa
Gong	2016	China	Asian	HB	479	203	181	235	63	79	96	28	TaqMan	0.953	0.329	LC
Yin	2018	China	Asian	PB	556	395	161	266	129	140	185	70	Illumina	0.889	0.346	LC
Wu	2019	China	Asian	HB	359	1190	134	185	40	422	560	208	TaqMan	4.072	0.044	HCC
Yang	2019	China	Asian	HB	431	431	152	213	66	171	190	70	PCR	0.372	0.542	BC
Yin	2018	China	Asian	HB	556	395	161	266	129	140	185	70	Illumina	0.889	0.346	LC
**6 Studies for *rs2735971* Poly morphism of the H19 Gene**																
							**AA**	**AG**	**GG**	**AA**	**AG**	**GG**				
Hua	2016	China	Asian	HB	1049	1396	43	302	704	46	422	928	TaqMan	2.128	0.145	BC
Li	2016	China	Asian	PB	1147	1203	773	334	40	765	398	40	TaqMan	0.278	0.598	CRC
Yang	2018	China	Asian	HB	472	472	12	126	327	13	139	313	KASP	0.001	0.974	HCC
Guo	2017	China	Asian	HB	461	739	129	141	191	80	308	351	Illumina	65.528	0.000	OSCC
He	2017	China	Asian	HB	193	383	11	94	88	32	182	169	TaqMan	4.848	0.028	Osteosarcoma
Li	2019	China	Asian	HB	200	200	10	62	128	4	70	126	TaqMan	0.479	0.489	BC
**8 Studies for *rs3024270* Poly morphism of the H19 Gene**																
							**CC**	**GC**	**GG**	**CC**	**GC**	**GG**				
Hua	2016	China	Asian	HB	1047	1395	174	527	346	260	688	447	TaqMan	1.254	0.263	BC
Li	2016	China	Asian	PB	1147	1203	385	527	235	420	582	201	TaqMan	4.860	0.027	CRC
Yang	2018	China	Asian	HB	472	472	95	225	151	81	215	170	KASP	0.449	0.503	HCC
Guo	2017	China	Asian	HB	362	740	75	183	104	145	350	245	Illumina	0.112	0.738	OSCC
He	2017	China	Asian	HB	193	383	17	91	85	31	179	173	TaqMan	1.134	0.287	OSC
Wu	2019	China	Asian	HB	359	1190	87	187	85	334	593	263	TaqMan	0.628	0.428	HCC
Yang	2019	China	Asian	HB	431	431	114	210	107	120	208	103	PCR	0.275	0.600	BC
Li	2019	China	Asian	HB	200	200	16	101	83	22	97	81	TaqMan	3.791	0.052	BC

**Abbreviation:** SOC: source of control; HB: hospital-based; HWE: Hardy–Weinberg equilibrium; BC: Bladder cancer; BCa: Breast cancer; LC: Lung cancer; HCC: Hepatocellular cancer; OSCC: Oral squamous cell carcinoma; OSC:Osteosarcoma; PC: Pancreatic cancer; CRC: Colorectal cancer; GC: Gastric cancer; OC: Ovarian cancer.

Meanwhile, we calculated the pooled ORs and 95% CIs using five genetic model in order to evaluate the affinity between lncRNA H19 ploymorphisms and cancer susceptibility, results of which were tabulated in **Table 2**. Also, stratification analysis by source of controls and genotypic method was applied to explore the heterogeneity of all studies.

**Table 2:** Meta-analysis results for the included studies of the association between LncRNA H19 polymorphisms and risk of cancer.

Variables	No. of studies	Allele model			Dominant model			Heterozygous model			Homozygous model				Recessive model		
		OR (95% CI)	P values	I-squared (%)	OR (95% CI)	P values	I-squared (%)	OR (95% CI)	P values	I-squared (%)	OR (95% CI)	P values		I-squared (%)	OR (95% CI)	P values	I-squared (%)
1. ***rs2839698 G>A***		A vs G			(GA+AA) vs GG			GA vs GG			AA vs GG				AA vs (GA+GG)		
All	16	1.08(0.99,1.19)	<0.001	75.3	1.08 (0.96,1.21)	<0.001	71.7	1.06 (0.95,1.17)	0.001	63.3	1.16 (0.92,1.42)	<0.001	71.7		1.13 (0.97,1.31)	0.002	58.1
Source of control																	
PB	2	**1.17 (1.04,1.31)**	0.347	<0.1	1.02 (0.68,1.54)	0.076	68.2	0.91 (0.53,1.55)	0.032	78.2	**1.41 (1.04,1.91)**	0.290	10.9		**1.46 (1.13,1.89)**	0.934	<0.1
HB	14	1.08 (0.97,1.19)	<0.001	76.9	1.09 (0.96,1.24)	<0.001	73.2	1.07 (0.95,1.20)	0.001	64.0	1.14 (0.92,1.42)	<0.001	73.1		1.08 (0.92,1.28)	0.004	57.9
Method of genotype																	
TaqMan	8	1.05 (0.96,1.14)	0.024	56.5	1.07 (0.96,1.18)	0.052	49.9	1.06 (0.96,1.17)	0.108	40.6	1.08 (0.89,1.32)	0.038	52.9		1.05 (0.88,1.26)	0.056	49.0
non-TaqMan	8	1.15 (0.95,1.39)	<0.001	84.2	1.12 (0.87,1.45)	<0.001	82.1	1.05 (0.84,1.33)	<0.001	75.9	1.34 (0.91,1.96)	<0.001	81.5		1.29 (0.95,1.76)	0.053	57.1
2. ***rs217727 G>A***		A vs G			(GA+AA) vs GG			GA vs GG			AA vs GG				AA vs (GA+GG)		
All	17	1.04 (0.96,1.13)	<0.001	69.9	1.07 (0.95,1.21)	<0.001	72.4	1.07 (0.94,1.21)	<0.001	71.9	1.06 (0.90,1.24)	0.001	59.2		1.03 (0.89,1.19)	0.001	59.7
Source of control																	
PB	4	0.97 (0.83,1.13)	0.051	61.4	0.90 (0.72,1.11)	0.048	62.1	0.85 (0.66,1.11)	0.014	71.6	0.97 (0.75,1.26)	0.154	42.9		1.06 (0.79,1.42)	0.056	60.4
HB	13	1.07 (0.97,1.18)	<0.001	70.4	**1.14 (0.99,1.30)**	<0.001	70.1	**1.15 (1.00,1.31)**	0.001	63.3	1.09 (0.90,1.31)	0.002	62.2		1.01 (0.85,1.20)	0.001	62.7
Method of genotype																	
TaqMan	9	1.04 (0.95,1.14)	0.009	60.8	1.11 (0.96,1.28)	0.001	70.4	1.12 (0.96,1.31)	<0.001	71.7	1.02 (0.84,1.22)	0.015	57.8		0.97 (0.81,1.17)	0.006	62.6
non-TaqMan	8	1.02 (0.86,1.21)	<0.001	76.9	1.01 (0.80,1.26)	<0.001	77.0	0.98 (0.77,1.24)	<0.001	75.5	1.11 (0.84,1.47)	0.018	58.8		1.12 (0.88,1.41)	0.044	51.6
3. ***rs2107425 C>T***		T vs C			(CT+TT) vs CC			CT vs CC			TT vs CC				TT vs (CT+CC)		
All	10	0.96 (0.89,1.04)	0.001	68.5	0.95 (0.85,1.06)	<0.001	71.5	0.95 (0.84,1.07)	<0.001	71.6	0.97 (0.83,1.13)	0.010	58.6		0.98 (0.87,1.12)	0.035	50.1
Source of control																	
PB	7	0.96 (0.88,1.06)	<0.001	75.8	0.92 (0.80,1.06)	<0.001	77.8	0.90 (0.78,1.03)	<0.001	75.5	1.01 (0.85,1.19)	0.016	61.5		1.04 (0.93,1.17)	0.190	31.2
HB	3	0.96 (0.82,1.13)	0.154	46.6	1.04 (0.88,1.23)	0.360	2.1	1.12 (0.94,1.32)	0.602	<0.1	0.85 (0.59,1.22)	0.114	54.0		0.79 (0.58,1.09)	0.150	47.3
Method of genotype																	
TaqMan	3	**0.86(0.80,0.94)**	0.396	<0.1	0.86 (0.71,1.05)	0.098	57.0	0.90 (0.68,1.21)	0.013	76.9	0.81 (0.62,1.04)	0.206	36.7		0.84 (0.59,1.20)	0.038	69.3
non-TaqMan	7	1.00 (0.91,1.10)	0.004	68.9	0.99 (0.86,1.13)	0.002	70.6	0.97 (0.85,1.11)	0.006	66.9	1.04 (0.87,1.24)	0.028	57.6		1.05 (0.96,1.15)	0.124	42.2
4. ***rs2735971 A>G***		G vs A			(AG+GG) vs AA			AG vs AA			GG vs AA				GG vs (AG+AA)		
All	6	0.91 (0.75,1.11)	<0.001	81.9	0.72 (0.44,1.17)	<0.001	86.8	0.68 (0.41,1.13)	<0.001	86.3	0.76 (0.45,1.29)	<0.001	81.5		0.99 (0.89,1.11)	0.247	26.2
Source of control																	
PB	1	0.89 (0.77,1.03)	-	-	**0.85 (0.71,1.00)**	-	-	**0.83 (0.70,0.99)**	-	-	0.99 (0.63,1.55)	-	-		1.05 (0.67,1.64)	-	-
HB	5	0.92 (0.72,1.19)	<0.001	85.5	0.69 (0.35,1.34)	<0.001	85.0	0.65 (0.33,1.29)	<0.001	84.4	0.71 (0.38,1.34)	<0.001	82.1		0.99 (0.88,1.11)	0.247	26.2
Method of genotype																	
TaqMan	4	0.96 (0.87,1.05)	0.503	<0.1	0.86 (0.66,1.12)	0.229	30.6	0.84 (0.63,1.12)	0.205	34.5	0.92 (0.66,1.30)	0.257	25.8		1.04 (0.91,1.19)	0.999	<0.1
non-TaqMan	2	0.82 (0.45,1.50)	<0.001	93.9	0.55 (0.16,1.86)	0.004	87.8	0.50 (0.15,1.67)	0.006	86.6	0.58 (0.18,1.90)	0.006	86.7		0.92 (0.77,1.10)	0.037	76.9
5. ***rs3024270 C>G***		G vs C			(CG+GG) vs CC			CG vs CC			GG vs CC				GG vs (CG+CC)		
All	8	1.03 (0.98,1.10)	0.237	24.1	1.07 (0.97,1.18)	0.630	<0.1	1.05 (0.95,1.17)	0.811	<0.1	1.09 (0.97,1.23)	0.218	21.4		1.03 (0.94,1.13)	0.177	31.5
Source of control																	
PB	1	1.11 (0.99,1.25)	-	-	1.06 (0.90,1.26)	-	-	0.99 (0.82,1.18)	-	-	**1.28 (1.01,1.61)**	-	-		1.28 (1.04,1.58)	-	-
HB	7	1.01 (0.95,1.08)	0.293	17.9	1.07 (0.95,1.20)	0.513	<0.1	1.09 (0.96,1.23)	0.809	<0.1	1.04 (0.90,1.19)	0.297	17.5		0.98 (0.88,1.08)	0.563	<0.1
Method of genotype																	
TaqMan	5	**1.08 (1.01,1.16)**	0.873	<0.1	**1.12 (1.00,1.26)**	0.788	<0.1	1.08 (0.96,1.22)	0.622	<0.1	**1.21 (1.05,1.39)**	0.851	<0.1		1.10 (0.99,1.23)	0.534	<0.1
non-TaqMan	3	0.93 (0.84,1.03)	0.309	14.9	0.95 (0.79,1.14)	0.532	<0.1	0.99 (0.82,1.20)	0.763	<0.1	0.88 (0.71,1.08)	0.353	4.1		0.88 (0.74,1.03)	0.404	<0.1

### rs2839698 G>A and cancer susceptibility

**Fig 2:**
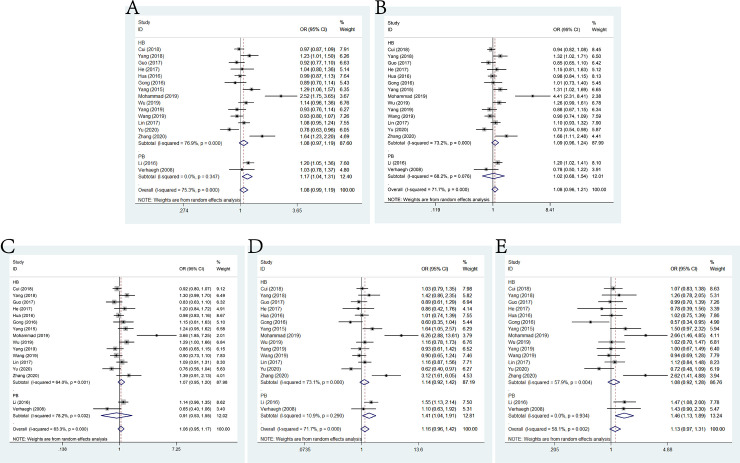
Forest plot of the association between H19 polymorphism rs2839698 G>A and cancer susceptibility. A: allele model; B: dominant model; C: heterozygote model; D: homozygote model; E: recessive model.

Sixteen studies about lncRNA H19 rs2839698 G>A ploymorphism and the susceptibility to cancer consisting 8872 cases and 11,723 controls met the inclusive criteria. The pooled ORs were 1.08 (95% CI: 0.99–1.19) for allele model, 1.08 (95% CI: 0.96–1.21) for dominant model, 1.06 (95% CI: 0.95–1.17) for heterozygote model, 1.16 (95% CI: 0.92–1.42) for homozygote model and 1.13 (95% CI: 0.97–1.31) for recessive model (**Fig 2**). Despite of no positive results, significant association between rs2839698 G>A and cancer susceptibility in population-based controls (allele model: OR = 1.17, 95% CI: 1.04–1.31; homozygote model: OR = 1.41, 95% CI: 1.04–1.91; recessive model: OR = 1.46, 95% CI: 1.13–1.89) was observed in the stratification analysis by source of control. In addition, no significant results were detected in the subgroup analysis by genotypic method.

### rs217727 G>A and cancer susceptibility

**Fig 3:**
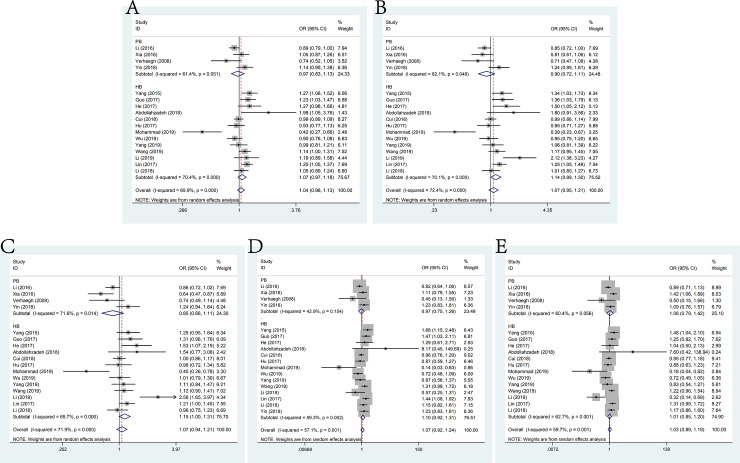
Forest plot of the association between H19 polymorphism rs217727 G>A and cancer susceptibility. A: allele model; B: dominant model; C: heterozygote model; D: homozygote model; E: recessive model.

In this meta-analysis, 17 Studies focusing on rs217727 G>A polymorphism and cancer susceptibility included 8678 cases and 11,207 controls. No significant association was indicated through the pooled risk estimation under allele model (OR = 1.04, 95% CI = 0.96–1.13), dominant model (OR = 1.07, 95% CI = 0.95–1.21), heterozygous model (OR = 1.07, 95% CI = 0.94–1.21), homozygous model (OR = 1.06, 95% CI = 0.90–1.24) and recessive model (OR = 1.06, 95% CI = 0.79–1.42) (**Fig 3**). While no significant results were observed in subgroup analysis by genotypic method, positive results were found in hospital-based controls (heterozygous model: OR = 1.15, 95% CI: 1.00–1.31).

### rs2107425 C>T and cancer susceptibility

**Fig 4:**
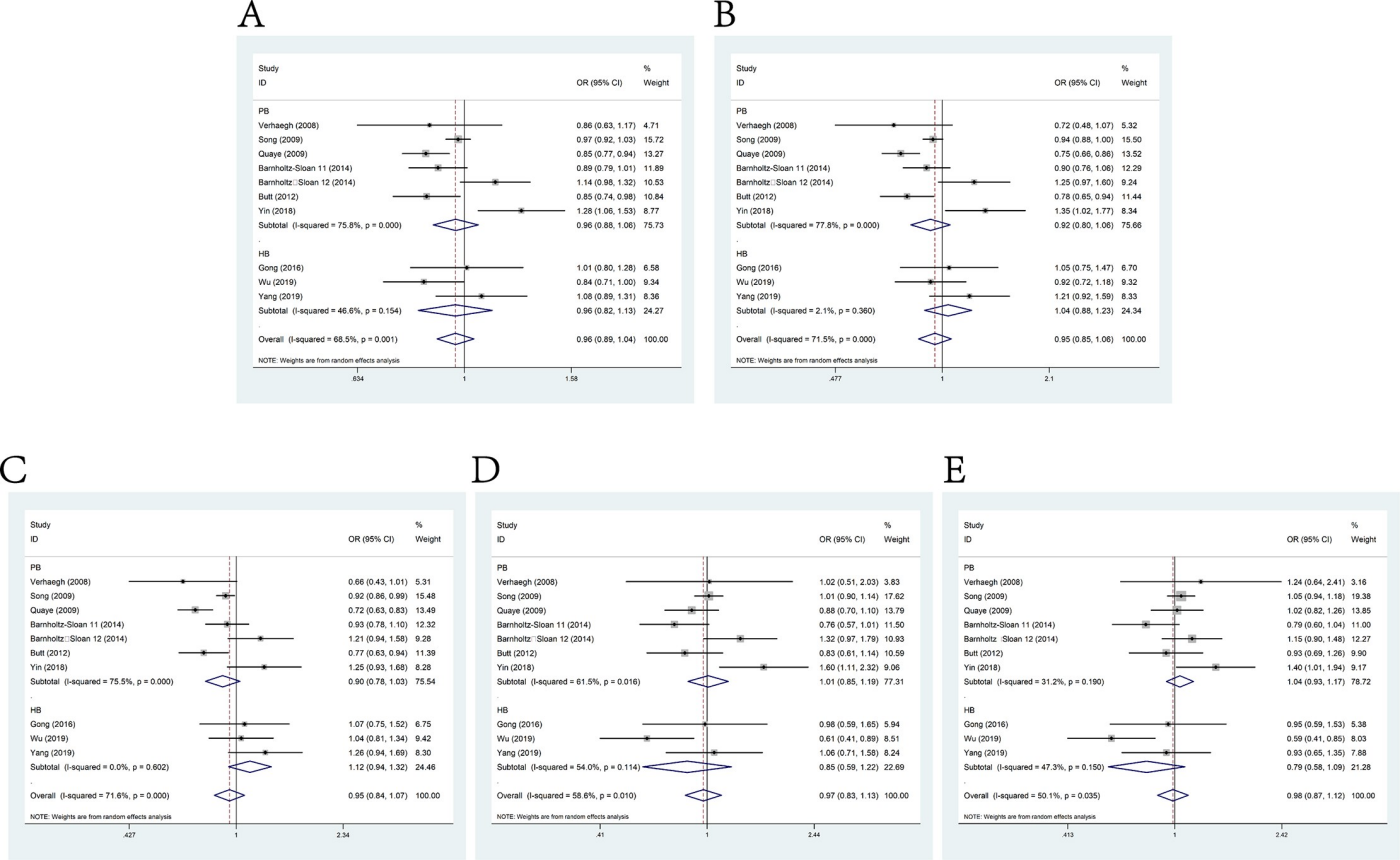
Forest plot of the association between H19 polymorphism rs2107425 C>T and cancer susceptibility. A: allele model; B: dominant model; C: heterozygote model; D: homozygote model; E: recessive model.

A total of 10 studies embodying 11,468 cases and 16,555 controls were investigated to LncRNA H19 polymorphic variants rs2107425 C>T and the susceptibility to cancer. Analogously, no significant association between rs2107425 C>T polymorphism and cancer risk was shown in the meta-analysis according to the pooled ORs of these studies under allele model (OR = 0.96, 95% CI = 0.89–1.04), dominant model (OR = 0.95, 95% CI = 0.85–1.06), heterozygous model (OR = 0.95, 95% CI = 0.84–1.07), homozygous model (OR = 0.97, 95% CI = 0.83–1.13) and recessive model (OR = 0.98, 95% CI = 0.87–1.12) (**Fig 4**). Nevertheless, as to the stratification analysis of genotypic method, the result was significant only in TaqMan (allele model: OR = 0.86, 95% CI = 0.80–0.94), while no significant results was detected in subgroup of source of control.

### rs2735971 A>G and cancer susceptibility

**Fig 5:**
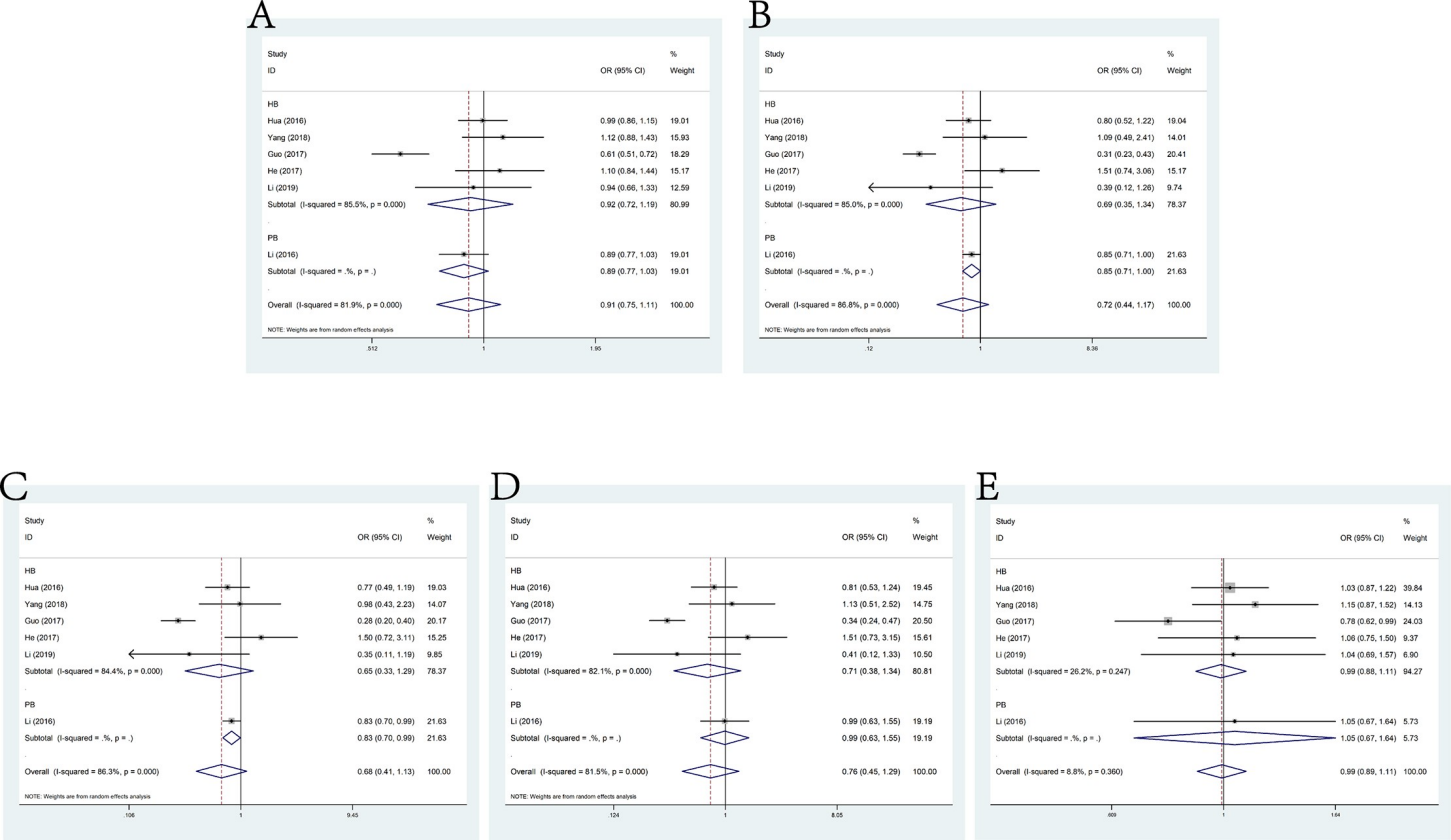
Forest plot of the association between H19 polymorphism rs2735971 A>G and cancer susceptibility. A: allele model; B: dominant model; C: heterozygote model; D: homozygote model; E: recessive model.

The present meta-analysis enrolled 3,522 cases and 4,393 controls from a sum of six studies on rs2735971 A>G polymorphism and cancer susceptibility. No significant association was observed among these studies under all the genetic models (allele model (OR = 0.91, 95% CI = 0.75–1.11), dominant model (OR = 0.72, 95% CI = 0.44–1.17), heterozygous model (OR = 0.68, 95% CI = 0.41–1.13), homozygous model (OR = 0.76, 95% CI = 0.45–1.29) and recessive model (OR = 0.99, 95% CI = 0.89–1.11)) (Fig 5). Additionally, the results of stratified analysis by genotypic method were not positive. By contrast, in subgroup analysis by source of control, feebly positive results were shown in population-based controls(dominant model: OR = 0.85, 95% CI = 0.71–1.00; heterozygous model: OR = 083, 95% CI = 0.70–0.99).

### rs3024270 C>G and cancer susceptibility

**Fig 6:**
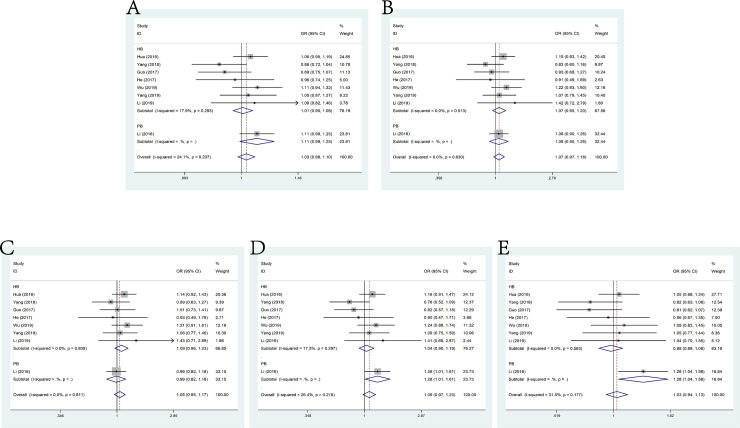
Forest plot of the association between H19 polymorphism rs3024270 C>G and cancer susceptibility. A: allele model; B: dominant model; C: heterozygote model; D: homozygote model; E: recessive model.

No significant association existed between rs3024270 mutation and cancer susceptibility as shown by the pooled risks of 8 relevant studies consisting 4,211 cases and 6,014 controls under allele model (OR = 1.03, 95% CI = 0.98–1.10), dominant model(OR = 1.07, 95% CI = 0.97–1.18), heterozygous model(OR = 1.05, 95% CI = 0.95–1.17), homozygous model (OR = 1.09, 95% CI = 0.97–1.23) and recessive model(OR = 1.03, 95% CI = 0.94–1.13) (**Fig 6**). However, stratification analysis by source of control indicated significant association with cancer susceptibility in the population-based control group (homozygous model: OR = 1.28, 95% CI = 1.01–1.61). Furthermore, the results of analysis stratified by genotypic method were more significant while using TaqMan than non-TaqMan methods (allele model: OR = 1.08, 95% CI = 1.01–1.16, dominant model: OR = 1.12, 95% CI = 1.00–1.26 and homozygous model: OR = 1.21, 95% CI = 1.05–1.39).

### Sensitivity analysis

**Fig 7:**
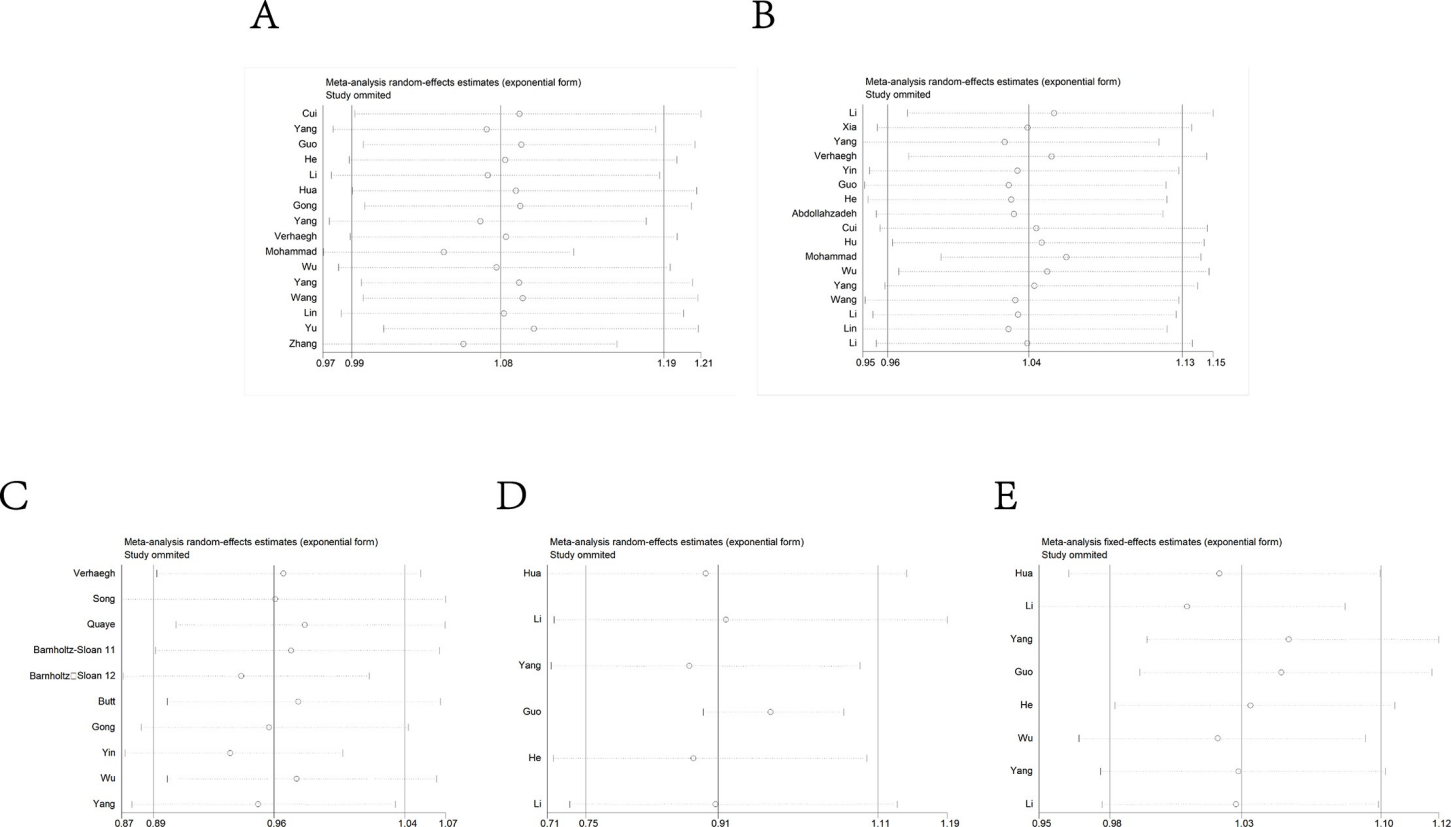
Sensitivity analysis under the allele model. A: rs2839698; B: rs217727; C: rs2107425; D: rs2735971; E: rs3024270.

Sensitivity analysis was carried out by removing single eligible study sequentially to detect individual study’s influence on the pooled results. According to the results, no single study was found affect the pooled OR in the allele model, suggesting a statistically robust results (**Fig 7**).

### Publication bias

**Fig 8:**
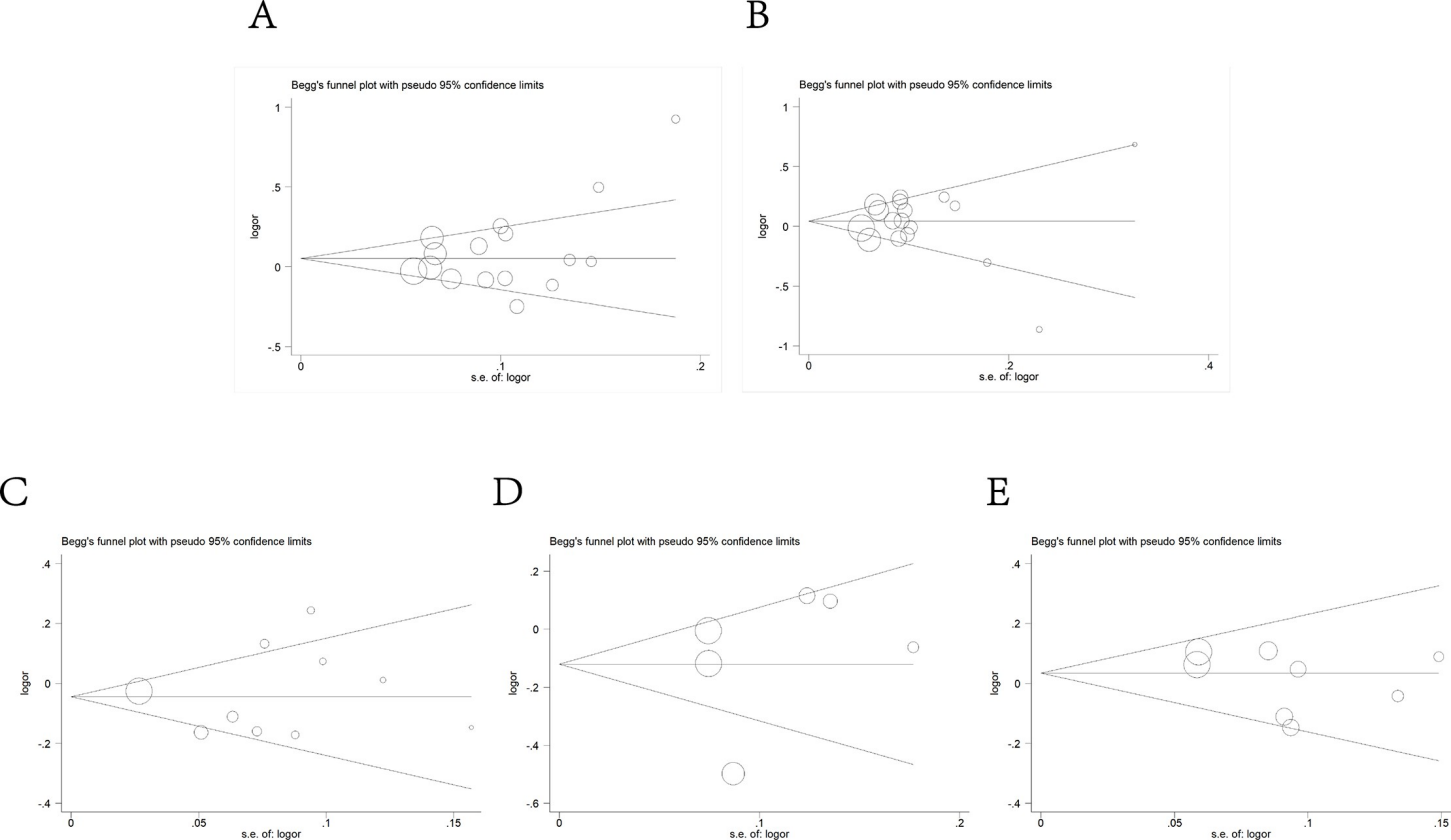
Begg’s funnel plot of publication bias test in the allele model. A: rs2839698; B: rs217727; C: rs2107425; D: rs2735971; E: rs3024270.

The Begg’s funnel plot and Egger’s test were utilized in the selected literature. With the symmetrical shapes of funnel plots shown in **Fig 8**, the absence of publication bias could be testified in the allele model (rs2839698: Begg’s Test *P* = 0.207 Egger’s test *P* = 0.169, rs217727: Begg’s Test *P* = 0.805 Egger’s test *P* = 0.943, rs2107425: Begg’s Test *P* = 0.421 Egger’s test *P* = 0.835, rs2735971: Begg’s Test *P* = 0.851 Egger’s test *P* = 0.593 and rs3024270: Begg’s Test *P* = 0.322 Egger’s test *P* = 0.305).

### Trial sequential analysis results

**Fig 9:**
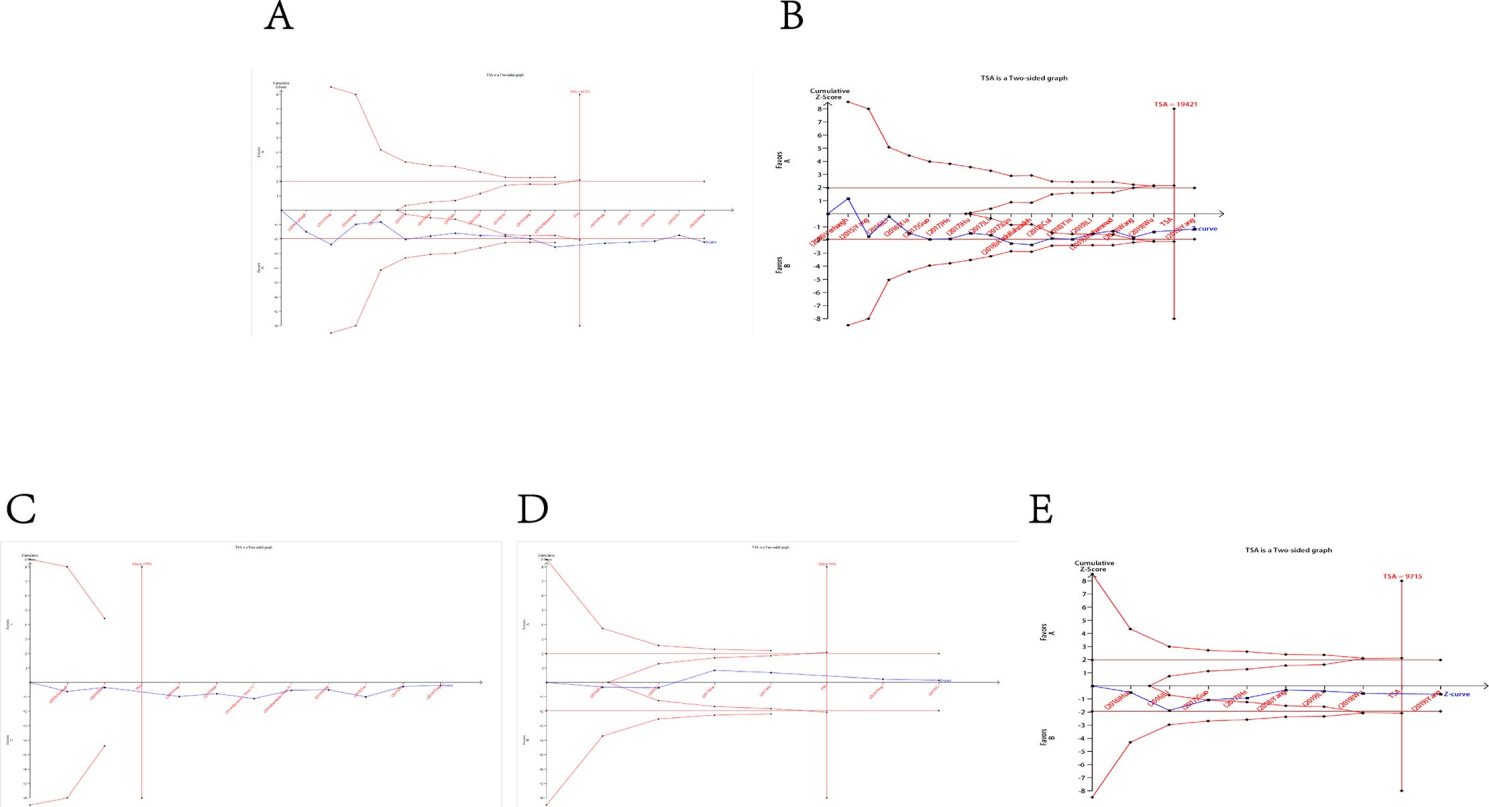
Trial sequential analysis of the association between H19 polymorphisms and the susceptibility to cancer. The required information size was calculated based on a 2-sided α = 5%, β = 15% (power 85%), and a relative risk reduction of 20%. A: rs2839698; B: rs217727; C: rs2107425; D: rs2735971; E: rs3024270.

In this meta-analysis, **Fig 9** showed that the cumulative Z-curve of all the H19 mutations investigated either crossed the trial sequential monitoring boundary or exceeded the required information size, indicating that the results about the associations between LncRNA H19 polymorphic variants (rs2839698 G>A, rs217727 G>A, rs2107425 C>T, rs2735971 A>G and rs3024270 C>G) and the susceptibility to cancer were firm evidence of effect.

## Discussion

An increasing number of studies have been focusing on the mutation of H19 when it comes to the genesis and development of various cancer [45]. As a long non-coding RNA, H19 lacks the open reading frame to translate protein whose end product is RNA sequence and can participate in downstream RNA regulatory [21, 46]. LncRNA H19 is an imprinted gene the aberrant expression of which is associated with cancer susceptibility [22]. In this meta-analysis, SNPs (rs2839698 G>A, rs217727 G>A, rs2107425 C>T, rs2735971 A>G and rs3024270 C>G) were included to investigate the relationship between these polymorphisms and the risk of cancer.

Previously, several meta-analysis on aberration of H19 have been conducted thanks to the identification of numerous LncRNAs [23, 24]. Though the results were contrary to many studies, a meta-analysis performed by Lv. revealed that rs217727 were uncorrelated to overall cancer risk [20]. It might account for the lack of the interactive microRNAs (miRNAs) which could influence the regulation and modification of lncRNAs SNPs directly [20, 47]. In that case, the position where gene structural changes caused by the polymorphism might differ from where the gene binds with elements such as miRNAs that regulate lncRNA expression, thus indicating no significant association with overall cancer risk. Also, another meta-analysis conducted by Li et al. inspired us on the possible reason [48]. The various cancer location and patient ethnicity might accounting for the discrepancies among the studies examined. In these studies, a small sample size and controversial results caused by the former factor might make these analysis relatively unreliable. Herein, we conducted this meta-analysis with the largest sample capacity and the most up-to-date studies and data, comprehensively analyzing all literatures to study such association. According to quantitative synthesis results, all the mutation mentioned above were found no significant association.

When stratified by source of control, significant association was found in the population-based control group between rs2839698, rs2735971 and rs3024270 polymorphisms and the susceptibility to cancer, whereas significant results in hospital-based control group were only found in SNP rs217727. Lack of the representativeness might account for the phenomena. Moreover, in the subgroup analysis by genotypic method, significant results were also found between the risk of cancer and rs2107425, rs3024270 polymorphisms adopting TaqMan method for genotyping, whereas similar results were not found while using other genotypic methods. The possible reason might be that different genotypic methods lead to different statistical results owing to their relative merits. The merits of TaqMan are the lower probability of PCR pollution due to that the reaction happens in the PCR process, avoiding separation and elution process [49].

TSA, as an statistical tool, is similar to interim analysis in a single trial, where trial monitoring boundaries are drawn for each outcome whether to continue additional trials to assess for evidence while a P value is small enough to show the projected effect or for futility [50]. The association shown in the results of this meta-analysis could be unreliable accounting for limited data. Therefore, TSA was adopted in order to diminish the probability of type I error and verify whether the evidence of our results was adequate or not. The results about the associations between LncRNA H19 polymorphic variants (rs2839698, rs217727, rs2107425, rs2735971 and rs3024270) and the susceptibility to cancer were firm evidence of effect [51]. Thus lager sample size for further verification is unnecessary.

Inevitably, several additional limitations should be warranted in this meta-analysis. (1). As a multifactorial disease, overall cancers are influenced by genetic combined with environmental factors. Focusing on single gene region, this meta-analysis ignored the complex interaction between various factors, in which case the association was unilateral; (2). The amount of studies in the subgroup analysis was relatively small. Subgroups with less than three studies were retained, thus might causing the potential false associations; (3). With the limit of the study amount, subgroup analysis based on race or cancer subtypes was not performed in this article. Additionally, subgroup analysis based on sex, age and gene dosage failed to be conducted accounting for the unavailability of relevant detailed data [52]. Besides, we failed to acquire the information of details such as age and gender distribution, amount of multiple gene mutation cases and so on, in which case, multi-trait analysis seems unable to implement [53]. (4). Quality control is also one of the limitations of our study. As most of the meta-analysis, the individual study quality determines overall quality. The test of Hardy–Weinberg equilibrium was conducted in this study, results of which indicates that genotype and allele frequencies remain unchanged over the generations. Nevertheless, specific quality test could be performed [54]. (5). Causal inference analysis could be another limitation. Gene mutation can affect the occurrence and development of cancer by affecting intermediate phenotype or other exposure factors. Previous study has shown that SNPs could be of much importance in modulating some novel biomarkers. Mendelian randomization study plays a vital role in discovering the causation of various cancers [55–57]. Conditions needs to be met for this study, while we failed to find the intermediate phenotype or other exposure factors in H19 mutation cases.

Genetic variability is significant when it comes to evaluation of disease susceptibility. In study by Allemailem at al., SNPs was helpful not only in diagnosis of prostate, but also in the further treatment for individuals [58]. Meanwhile, contributions have been made in plenty of studies retrieved in our article to testified the possibility of H19 SNPs in diagnosis and individualized treatment of various cancer. In this meta-analysis, we concentrated on the association between the overall cancer susceptibility and H19 mutation. Insufficient data of gene dosage and tumor staging from raw studies adds complications to establishing a prediction model [59, 60].

Consensus has been reached that H19 is involved in various biological process, but the potential mechanisms remain unknown. The study by Zheng at al. revealed that gene mutation can promote self adaptation [61]. On the other hand, current researches have focused on the N4-acetylcytidine on RNA, which can impact the development of cancer [62]. Thus, further exploration of according mechanism is necessary. Hence, to guaranty reliability of our meta-analysis, more large-sample, multi-center and high-quality researches should focus on the influence of different factors in the subsequent studies.

## Conclusion

To conclude, the results of this meta-analysis revealed that five H19 polymorphisms (rs2839698 G>A, rs217727 G>A, rs2107425 C>T, rs2735971 A>G and rs3024270 C>G) had no significant association with the overall cancer susceptibility, thereby suggesting that H19 might be not qualified for the ideal marker in the diagnosis and treatment of cancer. However, after the stratification analysis, inconsistent results still existed in different genotypic method and source of control. Thus, more high-quality studies on cancer patients of different factors were needed to confirm these findings.

## Supporting information

S1 Checklist PRISMA_2020_checklist. (DOCX)

S1 Data Raw data. (XLSX)
