# Supplementary material for: The influence of LncRNA H19 polymorphic variants on susceptibility to cancer: A systematic review and updated meta-analysis of 28 case-control studies
Source: PLoS One. 2021 Jul 26;16(7):e0254943. doi: 10.1371/journal.pone.0254943 (PMC8312943; doi:10.1371/journal.pone.0254943)
Supplement: S1 Checklist — (DOCX) [file pone.0254943.s001.docx]

| **Section and Topic** | **Item #** | **Checklist item** | **Location where item is reported** |
| --- | --- | --- | --- |
| **TITLE** | | |  |
| Title | 1 | The report is identified as a meta-analysis. | 1 |
| **ABSTRACT** | | |  |
| Abstract | 2 | The abstract contains Objective, Methods, Results and Conclusion. | 2 |
| **INTRODUCTION** | | |  |
| Rationale | 3 | Described in the Introduction. | 3 |
| Objectives | 4 | Stated in the Introduction. | 3 |
| **METHODS** | | |  |
| Eligibility criteria | 5 | Explained in the Methods. | 4 |
| Information sources | 6 | Specified in the Methods. | 4 |
| Search strategy | 7 | Presented in the Methods. | 4 |
| Selection process | 8 | Specified in the Methods. | 4 |
| Data collection process | 9 | Specified in the Methods. | 4 |
| Data items | 10a | Listed and defined in the Methods. | 4 |
|  | 10b | Listed and defined in the Methods. | 5 |
| Study risk of bias assessment | 11 | Specified in the Methods. | 5 |
| Effect measures | 12 | Specified in the Methods. | 5 |
| Synthesis methods | 13a | Described in the Methods. | 6 |
|  | 13b | Described in the Methods. | 4 |
|  | 13c | Described in the Methods. | 5 |
|  | 13d | Described in the Methods. | 6 |
|  | 13e | Described in the Methods. | 5 |
|  | 13f | Described in the Methods. | 5 |
| Reporting bias assessment | 14 | Described in the Methods. | 5 |
| Certainty assessment | 15 | Described in the Methods. | 5 |
| **RESULTS** | | |  |
| Study selection | 16a | Described in the Results. | 6 |
|  | 16b | Cited in the Results. | 6 |
| Study characteristics | 17 | Cited in the Results. | 7 |
| Risk of bias in studies | 18 | Presented in the Results. | 9 |
| Results of individual studies | 19 | Presented in the Results, and also presented as Table 1 and Figure2 to Figure 6. | 7 |
| Results of syntheses | 20a | Summarized in the Results. | 9 |
|  | 20b | Presented in the Results, and also described in the Table 2. | 7 |
|  | 20c | Presented in the Results. | 7 |
|  | 20d | Presented in the Results. | 9 |
| Reporting biases | 21 | Presented in the Results. | 9 |
| Certainty of evidence | 22 | Presented in the Results, and also described in the Table 2. | 7 |
| **DISCUSSION** | | |  |
| Discussion | 23a | Interpreted in the Discussion. | 9 |
|  | 23b | Discussed in the Discussion. | 11 |
|  | 23c | Discussed in the Discussion. | 11 |
|  | 23d | Discussed in the Discussion. | 10 |
| **OTHER INFORMATION** | | |  |
| Registration and protocol | 24a | The review was not registered. |  |
|  | 24b | A protocol was not prepared. |  |
|  | 24c | Nothing. |  |
| Support | 25 | No funding or financial support was received for this study. | 11 |
| Competing interests | 26 | We declare that we have no conflict of interest. | 12 |
| Availability of data, code and other materials | 27 | All of the data relevant in this study are publicly available with the attached information or online data base. | 12 |

*From:*  Page MJ, McKenzie JE, Bossuyt PM, Boutron I, Hoffmann TC, Mulrow CD, et al. The PRISMA 2020 statement: an updated guideline for reporting systematic reviews. BMJ 2021;372:n71. doi: 10.1136/bmj.n71

For more information, visit: <http://www.prisma-statement.org/>
