# Supplementary material for: The influence of LncRNA H19 polymorphic variants on susceptibility to cancer: A systematic review and updated meta-analysis of 28 case-control studies
Source: PLoS One. 2021 Jul 26;16(7):e0254943. doi: 10.1371/journal.pone.0254943 (PMC8312943; doi:10.1371/journal.pone.0254943)
Supplement: Attachment — Submitted filename: Response to Reviewers.doc [file pone.0254943.s003.doc]

Dear Editor/Reviewers：

Thank you for your prompt attention to our manuscript and helpful suggestions. We are pleased to follow your comments and the manuscript has been extensively revised according to your advice. Moreover, we have had our manuscript revised by an experienced colleague. In addition, we carefully proofread the manuscript to minimize typographical, grammatical, and bibliographical mistakes. The corresponding explanations of each point which is raised in your comments are as follows:

**Reviewer #1**

**Question 1:** The following papers can be cited and followed for the meta-analytic procedures (if the data is not enough available, at least DISCUSSION should be added as the LIMITATION of this study with enough cited references to support the viewpoints.

Ref 1: Wu Y, et al. Multi-trait analysis for genome-wide association study of five psychiatric disorders. Transl Psychiatry. 2020 Jun 30;10(1):209.

Ref 2: Jiang L, et al. Sex-Specific Association of Circulating Ferritin Level and Risk of Type 2 Diabetes: A Dose-Response Meta-Analysis of Prospective Studies..J Clin Endocrinol Metab. 2019 Oct 1;104(10):4539-4551.

Ref 3: Xu M, et al. Quantitative assessment of the effect of angiotensinogen gene polymorphisms on the risk of coronary heart disease. Circulation. 2007 Sep 18;116(12):1356-66.

Trans-ethnic and trans-trait meta-analysis can be referred to Ref 1.

Subgroup analyses based on cancer subtypes, sex, age, race, gene dosage can be referred to Ref 2.

Quality control can be referred to Ref 3.

**Authors’ response:** Thank you for your careful reading and helpful questions. In the previous literature study, a large number of article related to H19 polymorphisms with cancer risk were found. Therefore, we decided to use meta-analysis to put together these data in each literature for further analysis under the steps of subject determination, literature retrieval and screening, data extraction, statistical analysis and discussion. As to the study quality assessment issue, we have conducted test for HWE of each study in our meta-analysis, and results of which has been attached as supplement files in the updated **Table 1**. We have conducted the subgroup analysis on genotype and source of controls as shown in the article. However, with the limit of the study amount, subgroup analysis based on race or cancer subtypes was not performed in this article regretfully. Additionally, subgroup analysis based on sex, age and gene dosage failed to be conducted accounting for the unavailability of relevant detailed data. We sincerely hope that more large-sample, multi-center and high-quality researches could emerge, based on which, a systematically update could be done.

Wu Y, et al. Multi-trait analysis for genome-wide association study of five psychiatric disorders. Transl Psychiatry. 2020 Jun 30;10(1):209.

Jiang L, et al. Sex-Specific Association of Circulating Ferritin Level and Risk of Type 2 Diabetes: A Dose-Response Meta-Analysis of Prospective Studies..J Clin Endocrinol Metab. 2019 Oct 1;104(10):4539-4551.

**Question 2:**  I am wondering if the SNPs can be used for cancer prediction, and if the authors may follow the following references to construct prediction model for cancer or cancer subtypes based on the biomarkers.

Ref 3: Yu H, et al. LEPR hypomethylation is significantly associated with gastric cancer in males.Exp Mol Pathol. 2020 Oct;116:104493. I suggest the authors cite and follow the above paper to clearly describe ROC curve for the evaluating the performences.

Ref 4: Liu M, et al. A multi-model deep convolutional neural network for automatic hippocampus segmentation and classification in Alzheimer's disease.Neuroimage. 2020 Mar;208:116459.

If machine-learning/deep learning can not be used, please discuss as the LIMITATION of this study with enough cited references to support the viewpoints.

**Authors’ response:** Thanks for your considerate consideration. Indeed, describing ROC curve in order to get a specific assessment of whether the H19 SNPs could serve as a predictor of cancer is fairly attractive. Nevertheless, considering the lack of research amount upon specific cancer types, we were worried about the reliability of such data analysis. Instead of that, we referred to relevant literature and found that genetic variability is significant when it comes to evaluation of disease susceptibility. In study by Allemailem at al., SNPs was helpful not only in diagnosis of prostate, but also in the further treatment for individuals, which indicated that H19 SNPs also have the potential ability of predicting the cancer development. Meanwhile, contributions have been made in plenty of studies retrieved in our article to testified the possibility of H19 SNPs in diagnosis and individualized treatment of various cancer.

53	Allemailem, K. S. et al., Single nucleotide polymorphisms (SNPs) in prostate cancer: its implications in diagnostics and therapeutics. AM J TRANSL RES 13 3868 (2021).

**Question 3:** I suggest that at least one paragraph in the DISCUSSION section is needed to explore the potential biological mechanisms of the identified SNPs in LncRNA H19 that can be used for the prediction of cancers, including DNA repair, cancer-related immunodeficiency, RNA modifications , LncRNA regulations, which may explain how the five SNPs in LncRNA H19 are playing roles on the overall cancer or specific cancers.

It is well know that immunology regulations may affect cancer development and people reported that Immunodeficiency may promote adaptive alterations of host gut- and tissue-based microbiome. Therefore I would suggest the authors to discuss the relationship and the potential mechanisms among Immunodeficiency, clinic-pathological information,SNP biomarkers/ LncRNA H19 gene expression, and overall survival/development of cancers. The following paper can be referred to for this discussion:

Ref 5:Zheng S, et al. Immunodeficiency Promotes Adaptive Alterations of Host Gut Microbiome: An Observational Metagenomic Study in Mice. Front Microbiol. 2019 Nov 1;10:2415. doi: 10.3389/fmicb.2019.02415. eCollection 2019.

The mRNA modifications are potentially new insights into this biological basis,especially N4-Acetylcytidine on LncRNA H19 expression. The following papers clearly disclosed the RNA abnormal expressions are mediated through RNA modifications. The recent progress in N4-Acetylcytidine on RNA expression is also playing key role on the cancer development. I suggest the authors discuss this LncRNA H19 modifications/ N4-Acetylcytidine with their findings in the DISCUSSION section. The knowledge on cancer development needs to be updated.

Ref 6: Jin G, et al. The Processing, Gene Regulation, Biological Functions, and Clinical Relevance of N4-Acetylcytidine on RNA: A Systematic Review. Mol Ther Nucleic Acids. 2020. PMID: 32171170

In the Discussion Section, the limitations and strength should be discussed in detail with enough citation, as I recommended, supporting the viewpoints. If all the above-suggested additional analyses cannot be employed for technique reasons, please discuss as the LIMITATION of this study.

**Authors’ response:** Thank you for your careful and wise suggestions. According to your advice, we have carefully read your opinions and had a heated discussion by all co-authors so as to reach a unified point of view. After the discussion, we searched studies on the mechanisms of the the identified SNPs in LncRNA H19. With detailed reading, we acquired plenty of precious information and hint upon the potential mechanisms of how H19 SNPs indeed influence cancer development. The study by Zheng at al. performed a research of immunodeficiency mice, and assessment of the microbiome of their fecal samples revealed that gene mutation can promote self adaptation. As you mentioned in the advice, immunology regulations may affect cancer development by promoting adaptive alterations of host gut- and tissue-based microbiome. We would like to pay more attention to this direction as soon as we obtain relevant pathological information in detail. Additionally, current researches have also focused on the N4-acetylcytidine on RNA, which can impact the development of cancer. However, related experiments and researches have not been conducted on LncRNA frequently, especially on H19 SNPs. Thus, we felt the urge of filling the blank of this field and further exploration of according mechanism is necessary. Besides, some the figures are too big to uploaded, so we just uploaded their screenshots. But we could surely provide the original version if necessary.

54	Zheng, S. et al., Immunodeficiency Promotes Adaptive Alterations of Host Gut Microbiome: An Observational Metagenomic Study in Mice. FRONT MICROBIOL 10 2415 (2019).

55	Jin, G., Xu, M., Zou, M. & Duan, S., The Processing, Gene Regulation, Biological Functions, and Clinical Relevance of N4-Acetylcytidine on RNA: A Systematic Review. Mol Ther Nucleic Acids 20 13 (2020).

Yours sincerely

Chuanquan Tu

Department of Urology, The First People’s Hospital of Lianyungang, Lianyungang Clinical Medical College of Nanjing Medical University, Lianyungang, 222002, China.

2021-05-30
